# Proper Applications for Surveys as a Study Methodology

**DOI:** 10.5811/westjem.2016.11.32000

**Published:** 2016-12-05

**Authors:** Andrew W. Phillips

**Affiliations:** Stanford University, Department of Anesthesia, Division of Critical Care, Stanford, California

A survey instrument is any series of pre-defined questions intended to collect information from people, whether in person, by Internet, or any other media.^1^,^2^ Surveys are ubiquitous in health professions education research, used in approximately half of recently published articles,^3^ likely because of their low cost, relative speed, and (often misguided) perception that they are simple to use.

A survey instrument is merely the tool used for survey methodology, which encompasses the entire application of the survey instrument, such as selecting a sampling frame, maximizing the response rate, and accounting for nonresponse bias.^4^ The distinction is important because survey methodology is a research method like any of the various other methodology options (e.g. observational cohorts and randomized controlled trials), and there are specific situations for which a particular method is indicated or contraindicated.

The goal of this article is to provide guidance to researchers about when a survey is the appropriate methodology for a given research question. The importance of methodology choice is second only to choosing the primary research question itself. For comprehensive survey methodology reviews, readers are encouraged to review dedicated references.^1^,^2^,^5^ The rest of this article will address the fundamental question: When should I use a new survey?

## WHEN TO USE A NEW SURVEY (INDICATIONS)

The best use of survey methodology is to investigate human phenomena, such as emotions and opinions.^2^ These are data that are neither directly observable, nor available in documents. Moreover, a *new* survey instrument is only indicated when a prior instrument does not exist or is determined empirically to have insufficient validity and reliability evidence for the sampling frame of interest.^1^,^2^

When properly constructed, a survey—regardless of topic and whether exploring an emotion or opinion—has the equivalent rigor of a psychometric instrument.^5^,^6^ A psychometric instrument can even be used as a survey to explore emotion.

For example, the Maslach Burnout Inventory (MBI) was created to address the novel (at the time) construct of burnout.^7^ As a construct, burnout is a cohesive idea, explained by supportive ideas (subscales that represent domains), but *not* fully explained by observable data. Burnout is a human quality and so must be addressed by a survey.

Similarly, an opinion is a human quality and must be addressed by a survey, such as a preference for a product or teaching method. It is worth stressing that opinion surveys also require the same rigor as psychometric instruments.

## WHEN NOT TO USE A SURVEY (CONTRAINDICATIONS)

### (Relative) Contraindication #1: Observable or Recorded Data Already Exist

Using a survey when observable or recorded data exist is a relative contraindication because—although direct observation or a primary source is the most accurate method—sometimes a survey is the only practical way to obtain the data. A survey, however, should be the last resort because it is subject to interpretation and recall bias.

For example, daily activity (e.g. amount of time spent with patients versus a computer) is more accurately recorded by a third-party observer than self-reporting on surveys.^8^ If direct measurement is not a reasonable possibility, then frequent journal entries, which could be considered a repeated measures survey method, is the next best option. *Circulation* has a good decision tree for researchers studying physical activity, and the principles can be applied to any difficult-to-measure activity.^9^

Another example of observable data is how much students learned. Actual learning gains (i.e. learning something new) are not equivalent to learners’ opinions of their learning gains.^10^–^12^ Learners’ opinions are a real entity and sometimes important for a study question. However, researchers should not substitute a survey of learners’ opinions for tangibly measurable learning gains (e.g. test score improvements or patient outcomes) if the study question is about actual learning gains.

Survey methodology can also be used when it is unreasonable to obtain the primary records themselves. For example, a researcher may ask an office of medical education to complete a survey with data such as total number of residents, how their elective time is used, and how many residents required remediation. Although obtaining the primary documents for each of these questions would be best, it would likely be improbable to obtain the information from all of the different specialties. Thus, the graduate medical education office can complete the survey instrument for the researcher. However, it is important that the survey is completed *using the records*, not an individual’s recollection*.*

It bears repeating that a survey should be the *last resort* for observable and recorded data. One of the most common misuses of survey methodology is to obtain observable and recorded data.

### Alternative Approach: Use Direct Observation or Records When Possible

Researchers should carefully evaluate the most accurate way to measure the variable(s) of interest. Offices of medical education or the Association of American Medical Colleges, for example, can be primary sources for population data. Using the most accurate source for different questions within a study may require combining data from an external source and data from a survey.

Example: Straus CM et al. Medical student radiology education: Summary and recommendations from a national survey of medical school and radiology department leadership. J Am Coll Radiol. 2014.11(6):606–610.^13^

Note how Straus and colleagues surveyed radiology department chairs for opinions but requested numerical information (e.g. number of students matching in radiology each year) from records held by the offices of medical education.^13^

### Contraindication #2: A Pre-Existing Survey Exists

Often a similar—if not exactly the same—concept has been surveyed by other researchers. Although the primary research question may warrant a survey methodology, a suitable existing survey is a contraindication to create and apply a new survey.* We as researchers are limiting greater concept understanding because we cannot combine findings, such as in a meta-analysis, 14 if we do not use pre-existing surveys when they are available. The Figure contains a list of resources to find pre-existing survey instruments.

### Alternative Approach

An early search for pre-existing surveys is essential if a researcher plans to use survey methodology. Use the exact same survey—word for word—if possible, and investigate reliability and validity evidence in the new cohort of interest, even if the exact same survey is used (word for word).^2^,^15^

Example: Galan F et al. Burnout risk in medical students in Spain using the Maslach Burnout Inventory-Student survey. Int Arch Occup Environ Health. 2011.84:453–459.^16^

Galan and colleagues defend their need to alter individual words for what they believed to be a unique cohort and successfully re-demonstrated reliability and validity evidence before using the survey.

### Contraindication #3: The Concept Is Ill-Defined

Survey methods range from a researcher personally asking respondents each question—with great ability to further explore respondent answers—to third-party questionnaires—without any ability to explore or clarify respondent answers. It is important to recognize the differences in data obtained from each survey format and apply the methodology appropriately. An ill-defined concept is a contraindication to use a survey, and qualitative grounded theory interviews or ethnography should be strongly considered. This especially applies to designing potential responses for survey questions.^2^

Researchers who use a questionnaire for a poorly defined concept run the risk of omitting options that respondents would have selected if they had been available because a questionnaire limits response options.† The results become artificially narrow and do not adequately represent the sampling frame.

### Alternative Approach

A questionnaire limits response options and should only be used when a concept is understood well enough to supply a full range of response options. Researchers should start with qualitative method interviews or focus groups^17^ to explore a wide range of concept interpretations and opinions.^2^

Example: McLeod et al. Using focus groups to design a valid questionnaire. Academic Medicine. 2000. 75(6):671.^18^

The authors in this example set out to explore a concept that had been previously overlooked. Since no prior data existed, they started with focus groups to first define the construct, then built a questionnaire to explore the construct in the cohort of interest.^18^

### Contradiction #4: The Sampling Frame Is Not Qualified

The accuracy of a survey is only as strong as the accuracy that each respondent can provide. Although a survey method may be indicated, it may be contraindicated in a certain sampling frame. For example, the meaning of learner evaluations of faculty has long been questioned. Are learners qualified to judge instructors? Are instructor evaluations by learners meaningful?^19^,^20^ Researchers who assert that learners are not qualified to evaluate instructors would also assert that a class survey about an instructor’s abilities would be inappropriate (although this practice is ubiquitous).

Another example of an unqualified sampling frame is when speculative questions are asked, such as, “What do your peers think?” Although a different context, the underlying principle remains the same since respondents are unqualified to present data for what others may think.

### Alternative Approach

Consider the qualifications of a given sampling frame for the particular question of interest. If the primary research question requires the respondents to have expertise, consider a sampling frame with that specific expertise or use a different study methodology, such as observation or testing.

Example: Grover PL. Evaluation of instructional skills of medical teachers: the participant observer in the medical school. Med Educ. 1980; 14:12–15.^21^

Grover introduces the idea of a trained third-party observer to evaluate medical student instructors. Depending on the primary research question (opinion of lecturing abilities versus learning outcomes), student examinations may be more accurate as well.

## CONCLUSION

Survey methodology is an important medical education research tool but should mainly be used to characterize unobservable, human phenomena such as emotions and opinions. Researchers should use methods other than surveys to gather observable data whenever possible. Moreover, many research questions are well suited to using mixed methods that include a survey in addition to other data collection methods.

## Figures and Tables

**Figure:**
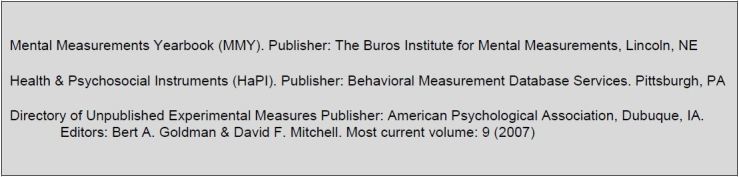
Databases of previously established survey instruments.

## Footnotes

* A more in-depth explanation of using pre-existing surveys is beyond the scope of this article, but researchers should be aware that even pre-existing surveys must be re-evaluated for validity and reliability evidence. Lack of validity and reliability evidence for a pre-existing survey when applied to a new sampling frame is an indication to edit the pre-existing survey or create a new one.

† Including “other” with a narrative response as a response option is not the best way—and usually still inadequate—to capture poorly understood concepts.
